# Overall Survival and Prognostic Factors in Metastatic Triple-Negative Breast Cancer: A National Cancer Database Analysis

**DOI:** 10.3390/cancers16101791

**Published:** 2024-05-08

**Authors:** Meghana Kesireddy, Lina Elsayed, Valerie K. Shostrom, Priyal Agarwal, Samia Asif, Amulya Yellala, Jairam Krishnamurthy

**Affiliations:** 1 Division of Hematology and Medical Oncology, Department of Internal Medicine, University of Nebraska Medical Center, Omaha, NE 68198, USAjairam.krishnamurthy@unmc.edu (J.K.); 2 Department of Biostatistics, University of Nebraska Medical Center, Omaha, NE 68198, USA

**Keywords:** metastatic triple-negative breast cancer, overall survival, prognostic factors, national cancer database, demographic factors, clinicopathological factors, treatment factors, cox proportional hazard model, multivariate analysis

## Abstract

**Simple Summary:**

Metastatic triple-negative breast cancer is an aggressive cancer with an average survival of 8 to 13 months. However, survival varies significantly among patients. We studied a large group of women in the National Cancer Database to better understand these survival differences. We found that the key factors influencing survival were comorbidity score (overall health status), histology (cancer subtype), number of metastatic sites (extent of cancer spread), and whether or not they received chemotherapy or immunotherapy. Our study’s findings can help better predict survival based on an individual’s specific factors and guide treatment strategies.

**Abstract:**

Background: Metastatic triple-negative breast cancer (TNBC) is aggressive with poor median overall survival (OS) ranging from 8 to 13 months. There exists considerable heterogeneity in survival at the individual patient level. To better understand the survival heterogeneity and improve risk stratification, our study aims to identify the factors influencing survival, utilizing a large patient sample from the National Cancer Database (NCDB). Methods: Women diagnosed with metastatic TNBC from 2010 to 2020 in the NCDB were included. Demographic, clinicopathological, and treatment data and overall survival (OS) outcomes were collected. Kaplan–Meier curves were used to estimate OS. The log-rank test was used to identify OS differences between groups for each variable in the univariate analysis. For the multivariate analysis, the Cox proportional hazard model with backward elimination was used to identify factors affecting OS. Adjusted hazard ratios and 95% confidence intervals are presented. Results: In this sample, 2273 women had a median overall survival of 13.6 months. Factors associated with statistically significantly worse OS included older age, higher comorbidity scores, specific histologies, higher number of metastatic sites, presence of liver or other site metastases in those with only one metastatic site (excluding brain metastases), presence of cranial and extra-cranial metastases, lack of chemotherapy, lack of immunotherapy, lack of surgery to distant sites, lack of radiation to distant sites, and receipt of palliative treatment to alleviate symptoms. In the multivariate analysis, comorbidity score, histology, number of metastatic sites, immunotherapy, and chemotherapy had a statistically significant effect on OS. Conclusions: Through NCDB analysis, we have identified prognostic factors for metastatic TNBC. These findings will help individualize prognostication at diagnosis, optimize treatment strategies, and facilitate patient stratification in future clinical trials.

## 1. Introduction

Breast cancer is the most commonly diagnosed cancer globally, surpassing lung cancer [1]. Triple-negative breast cancer (TNBC) accounts for 15–20% of all breast cancers [2]. TNBCs are estrogen and progesterone receptor-negative, <1% by immunohistochemistry (IHC), and human epidermal growth factor receptor 2 (HER2) negative (0–1+ by IHC or 2+ by IHC with FISH negative). TNBC is more commonly diagnosed in women younger than 40 (odds ratio of 2.13, 95% CI 1.34–3.39) and African–American women (odds ratio 2.41, 95% CI 1.81–3.21) compared with those over 50 and Caucasian women, respectively [3]. 

Compared with hormone receptor-positive or HER2-positive breast cancers, TNBC is known for its aggressiveness with larger tumor size, lymph node involvement, higher tumor grade, higher relapse rate of localized TNBC to metastatic TNBC, and a propensity to affect younger patients [3,4,5]. Individuals with TNBC experience an increased likelihood of distant recurrence within 5 years of diagnosis, with a tendency toward visceral as opposed to bony sites of relapse [6,7,8,9]. Due to the lack of biomarkers for targeted therapies like hormone receptor or HER-2 expression, there are only a few approved agents for TNBC, and the treatment mainly involves cytotoxic agents [10,11]. All these factors contribute to reduced overall survival in TNBC compared to other types of breast cancer, in both localized and metastatic settings. The 5-year overall survival (OS) for metastatic TNBC is around 11%, with a median OS of around 11 to 13 months [5,12]. Despite this bleak prognosis, considerable prognostic heterogeneity exists at the individual patient level, with a wide range of OS from 0.8 months to 99.8 months [5,13].

Survival estimation based on results observed in therapeutic clinical trials fails to offer a realistic prognosis. Also, discussing the median OS with patients without considering their individual factors is not appropriate, especially in metastatic TNBC, due to significant survival heterogeneity at the patient level. To address this gap and provide clinicians with personalized prognostic insights, our study aims to explore the impact of demographic, clinicopathological, and treatment factors on OS, utilizing a large patient sample from the National Cancer Database (NCDB). 

## 2. Methods

This study utilized the National Cancer Database (NCDB), which includes data from over 1500 Commission on Cancer (CoC) programs that treat approximately 70% of all newly diagnosed cancers in the United States [14,15]. A retrospective review of de-identified patient data adhering to the established procedures for NCDB analyses was conducted and, hence, this study was exempt from our institutional review board. Demographics (age, race, and Charlson-Deyo comorbidity score), clinicopathological variables (histology, HER2 IHC expression, number of metastatic sites, and sites of metastases), and treatment variables (first-line chemotherapy, immunotherapy, days from diagnosis to systemic therapy, distant site surgery, distant site radiation, and palliative treatment to alleviate symptoms) were selected for analysis. A total of 2273 women diagnosed with metastatic TNBC between 2010 and 2020 were included, with all the required information on the variables analyzed in our study. 

Charlson-Deyo comorbidity scores were divided into 4 groups: score 0, score 1, score 2, and score 3 or more. Histology included invasive ductal carcinoma, invasive lobular carcinoma, adenocarcinoma with metaplasia, adenocarcinoma not otherwise specified (NOS), and other carcinomas. Other carcinomas predominantly included carcinoma NOS, small-cell carcinoma, neuroendocrine carcinoma, non-small-cell carcinoma, and inflammatory carcinoma, as well as adenoid cystic and cribriform carcinoma and mucoepidermoid carcinoma. The number of metastatic sites refers to the number of organs involved with metastatic cancer. Time to initiation of systemic therapy initiation was categorized as <2 weeks, 2–4 weeks, >4 weeks, or no systemic therapy. Palliative treatment refers to any treatment aimed to relieve symptoms, which may have involved surgery, radiation, systemic therapy, and/or other pain management. The overall survival (OS) was defined as the time from the diagnosis of metastatic TNBC until death.

Demographic, clinicopathological, and treatment data were described using frequencies and percentages. Kaplan–Meier (KM) curves were used to estimate the OS for each variable of interest. The log-rank test was used to identify OS differences between groups for each variable in the univariate analysis. The Cox proportional hazard model with backward elimination was used to identify factors affecting the OS in the multivariate analysis. The median OS in months, adjusted hazard ratios, and 12-month survival estimates with 95% confidence intervals (CI) were reported. PC SAS version 9.4 was used for all analyses. The statistical significance level was set at 0.05 for all analyses. 

## 3. Results

A total of 2273 women diagnosed with de novo metastatic TNBC had a median OS of 13.6 months (95% CI 12.81–14.65). Table 1 details demographic, clinicopathologic, and treatment variables. Most women were aged 41–70 (63.1%), Caucasian (67.4%), and had a Charlson-Deyo comorbidity score of 0 (76.2%). The majority had invasive ductal carcinoma (81%), HER-2 IHC expression of 0 (53.5%), extra-cranial metastases only (89%), and a single metastatic site (49.7%). The majority of women received multi-agent chemotherapy as first-line therapy (41.6%), no immunotherapy (80.4%), had >4 weeks from diagnosis to initiation of systemic therapy (49.8%), no surgery or radiation to the distant lymph node or metastatic site (93.9% and 70.5% respectively), and received no palliative treatment to alleviate symptoms (75.7%).

Table 1 details the median OS, log-rank *p*-value, hazard ratio (HR), and 12-month survival estimates of different groups for each demographic, clinicopathological, and treatment variable in the univariate analysis. 

In regard to the demographic variables, the OS was significantly worse for the 71–90 age group, with the shortest median OS of 9.6 months, compared with the 19–40 age group (HR 1.52, 95% CI 1.24–1.85); the OS was similar in the 19–40 (median OS 15.5 months) and 41–70 age groups (median OS 15.6 months). Charlson-Deyo comorbidity score groups of 1 (HR 1.22, 95% CI 1.06–1.41), 2 (HR 1.8, 95% CI 1.47–2.22), and 3 or more (HR 2.46, 95% CI 1.94–3.12) were associated with statistically significantly worse OS compared with the score 0 group, demonstrating a clear trend of decreasing survival as the comorbidity score increased. Women with a score of 3 or more had the shortest median OS of 4.6 months compared with the longest median OS of 15.3 months in the score 0 group. No significant differences in OS were observed based on race (*p* = 0.2033). 

Among the cancer related/clinicopathological variables, OS was significantly worse for adenocarcinoma NOS, with a median OS of 4 months (HR 1.89, 95% CI 1.51–2.37), and the group of other carcinomas, with a median OS of 6.7 months (HR 1.83, 95% CI 1.55–2.17), compared with the invasive ductal carcinoma group. However, OS was similar among the invasive ductal carcinoma (median OS 15.2 months), invasive lobular carcinoma (median OS 12 months), and adenocarcinoma with metaplasia (median OS 14 months) groups. Regarding the numbers of metastatic sites, patients with two (HR 1.72, 95% CI 1.53–1.94), three (HR 1.99, 95% CI 1.72–2.3), four (HR 3.16, 95% CI 2.62–3.8), and five or six (HR 4.61, 95% CI 3.47–6.13) metastatic sites had statistically significantly worse OS compared with only one metastatic site, with a clear trend of decreasing survival as the number of metastatic sites increased. Women with five or six metastatic sites had the shortest median OS of 2.6 months compared with the longest median OS of 19.8 months in patients with a single metastatic site. In the subgroup analysis of those with only a single metastatic site (excluding brain-only metastases due to their known worse prognosis), women with bone metastases only (HR 1.36, 95% CI 1.08–1.72), liver metastases only (HR 1.67, 95% CI 1.29–2.17), or other site metastases only (HR 1.85, 95% CI 1.34–2.55) had worse OS compared with those with lymph node metastases only, while the OS was similar in those with lymph node metastases only and lung metastases only. The OS was statistically significantly worse in those with both cranial and extra-cranial metastases, with the shortest median OS of 5.3 months compared with cranial metastases only (HR 1.7, 95% CI 1.16–2.49), and OS was similar in those with cranial metastases only (median OS 11.8 months) and extra-cranial metastases only (median OS 14.6 months). No significant differences in OS were observed based on HER2 IHC expression (*p* = 0.3099). 

Regarding the treatment variables, OS was statistically significantly better for women who received either single-agent first-line chemotherapy, with a median OS of 16.4 months (HR 0.29, 95% CI 0.25–0.33), or multi-agent first-line chemotherapy, with a median OS of 19.9 months (HR 0.23, 95% CI 0.21–0.26), compared with no chemotherapy, with a median OS of 2.9 months. This improvement in OS with first-line chemotherapy remained consistent across all the Charlson-Deyo comorbidity score groups in the sub-group analysis; refer to Table 2 for details. Women who received immunotherapy had better OS, with a median OS of 25.6 months compared with the median OS of 11.5 months in the no-immunotherapy group (HR 0.49, 95% CI 0.42–0.56). Regarding the time from diagnosis to initiation of systemic therapy, women who waited >4 weeks had a better median OS of 20.6 months (HR 0.21, 95% CI 0.18–0.23), followed by those waiting 2–4 weeks who had a median OS of 15.6 months (HR 0.26, 95% CI 0.23–0.31) and those waiting <2 weeks, with a median OS of 10.8 (HR 0.34, 95% CI 0.28–0.43), compared with women receiving no systemic therapy who had a median OS of 2.7 months. 

Surgery of the distant lymph node or distant metastatic site was associated with significantly better OS, with a median OS of 29.2 months, compared with no surgery, with a median OS of 13.1 months (HR 0.5, 95% CI 0.38–0.66). Similarly, women who received radiation to the distant site had a significantly better OS, with a median OS of 14.7 months, compared with no radiation, with a median OS of 12.6 months (HR 0.86, 95% CI 0.76–0.98). However, the receipt of palliative treatment to alleviate symptoms was associated with significantly worse OS, with a median of 11.3 months, compared with no palliative treatment, with a median OS of 14.5 months (HR 1.18, 95% CI 1.06–1.32). 

**Figure 1:**
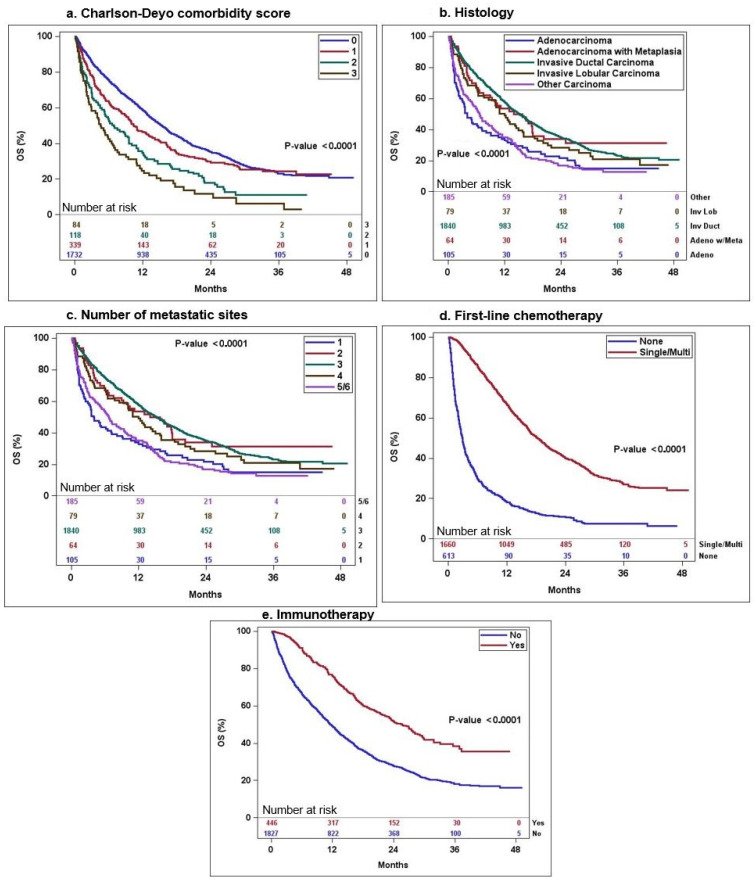
Kaplan–Meier survival curves of the five significant variables in the multi-variate analysis.

Based on the results from the univariate analysis, the following variables were included in the final multivariate analysis: age, Charlson-Deyo comorbidity score group, histology, number of metastatic sites, site of metastases: cranial only/cranial and extra-cranial/extra-cranial only, receipt of first-line chemotherapy, receipt of immunotherapy, and receipt of palliative treatment to alleviate symptoms. In the multi-variate Cox proportional hazard analysis, the Charlson-Deyo comorbidity score, histology, number of metastatic sites, receipt of first-line chemotherapy, and receipt of immunotherapy were identified as the five factors independently influencing OS, as detailed in Table 3. Figure 1 displays the univariate Kaplan–Meier survival curves for these five prognostic factors. The variables of surgery and radiation of the distant site were excluded from the multi-variate analysis, despite significance in the univariate analysis, due to missing information from some patients. Similarly, the time from diagnosis to initiation of systemic therapy was also excluded as a variable, due to its overlapping subgroups with variable of first-line chemotherapy. 

## 4. Discussion

Prognostication based on the results observed in therapeutic clinical trials may not realistically estimate prognosis in the real world. Fewer comorbidities and better functional status due to stringent eligibility criteria, lower cancer burden without visceral crisis or severe symptoms, better treatment compliance, and increased access to healthcare and other supportive resources typically seen in participants lead to longer OS in clinical trials [16,17,18]. A meta-analysis of three phase III trials of first-line treatment in metastatic TNBC reported a median OS of 17.5 months, surpassing the real-world OS estimates of 11 to 13 months [5,12,18,19]. These reported real-world studies with limited sample size and selective representation may not accurately reflect the diverse TNBC patient population in the United States. Our study, the largest to date, included 2273 metastatic TNBC women from the NCDB, encompassing data from multiple institutions treating over 70 percent of all cancer patients in the United States. It revealed a median OS of 13.6 months, consistent with prior reports. 

We identified five independent prognostic variables for survival in metastatic TNBC in the multivariate analysis: Charlson-Deyo comorbidity score, histology, number of metastatic sites, receipt of first-line chemotherapy, and receipt of immunotherapy. Affirming the crucial role of overall health in prognosis, there was an inverse correlation between Charlson-Deyo comorbidity score and OS. This finding was consistent with a prior study of non-metastatic TNBC that showed those with a Charlson comorbidity index score of ≥3 had a significantly higher risk of death compared with those without comorbidities among African–American patients, and comorbidities at diagnosis increased risk of death independent of TNBC [20]. In the metastatic setting, our study demonstrated that histology is an independent prognostic factor. Invasive ductal carcinoma, invasive lobular carcinoma, and adenocarcinoma with metaplasia had similar OS, while the adenocarcinoma NOS and other carcinoma groups (encompassing aggressive histologies like inflammatory carcinoma, neuroendocrine carcinoma, and small-cell carcinoma) exhibited worse OS. In non-metastatic TNBC, prior studies showed that invasive lobular carcinoma and metaplastic carcinoma were associated with worse OS compared with invasive ductal carcinoma [21,22]. However, another study adjusting for tumor stage and demographics found similar OS between invasive lobular carcinoma and invasive ductal carcinoma [23], highlighting the OS differences between invasive lobular carcinoma and invasive ductal carcinoma in previous studies might be due to potential selection bias and inadequate adjustment for clinically relevant covariates. In our study, there was an inverse correlation between the number of metastatic sites and OS, suggesting that the number of metastatic sites may reflect disease burden. This finding was consistent with a prior study of TNBC that showed a higher risk of death for those with more than one metastatic site compared with those with only one metastatic site [12]. Receipt of first-line chemotherapy, either single-agent or multi-agent, the primary backbone of treatment for metastatic TNBC, was an independent prognostic factor for OS, as those not receiving experienced worse OS that was probably due to faster cancer progression. Receipt of immunotherapy was also an independent prognostic factor, associated with better OS, consistent with the results from Impassion 130 and Keynote 522 trials. In 2019, atezolizumab, a PD-L1 inhibitor, was approved as a first-line treatment in combination with chemotherapy for PD-L1 ≥ 1% tumors, based on the Impassion130 trial but was later withdrawn in 2021 due to a lack of PFS and OS improvement in the Impassion 131 trial [24,25]. In 2020, pembrolizumab, a PD-1 inhibitor, was approved as first-line treatment in combination with chemotherapy in PD-L1 ≥ 10% tumors, based on the Keynote 355 trial [26]. PD-L1 positivity in TNBC is approximately 20–47% [27,28]. In our study, which included patients from 2010–2020, 19.6% of total patients had received immunotherapy, some probably in later lines after having received other treatments before the immunotherapy approval. 

In our study, poor OS was observed in the 71–90 age group in the univariate analysis, but age was not an independent prognostic factor in the multi-variate analysis. A prior study on metastatic TNBC surprisingly revealed a poor prognosis in those under 50 years old [5]. Studies in non-metastatic TNBC examining the impact of age at diagnosis on OS yielded conflicting results [29,30,31]. Age itself may not be an independent prognostic factor, but advanced age is usually linked to a higher burden of comorbidities, poor overall health, and potentially lower chemotherapy uptake, adversely affecting OS [16]. Thus, treatment decisions should consider functional age over chronological age [32]. While OS was worse for those with both cranial and extra-cranial metastases according to the univariate analyses, the location of metastases (cranial only vs. cranial and extra-cranial vs. extra-cranial only) lost significance in the multi-variate analyses. TNBC typically has a higher incidence of brain metastases, at 30% with a median OS of 5 months after brain metastases [33,34]. Factors impacting mortality in brain metastases include having more than three brain lesions, symptomatic brain metastases, lack of brain metastases-directed treatment, and uncontrolled extra-cranial metastases [35]. It is possible that the location of metastases did not emerge as an independent prognostic factor in our study due to the small number of patients with cranial metastases (only 2% had cranial metastases only and 9% had both cranial and extra-cranial metastases). Alternatively, overall cancer burden and ability to receive treatment (both systemic therapy and brain metastases-directed local treatments like radiation) may be the clinically relevant confounding covariates impacting OS, rather than the presence of brain metastases. Palliative treatment (to alleviate symptoms), despite showing an association with poor OS in the univariate analysis, was not identified as an independent prognostic factor in the multi-variate analysis, as it probably reflected a higher disease burden rather than treatment impact. 

In the subgroup analysis of patients with a single metastatic site (excluding brain-only metastases, due to their known worse prognosis) [33,35], those with lung metastases only or distant lymph node metastases only demonstrated better OS, indicating potential prognostic implications of the site of metastatic disease in this subgroup that warrant further evaluation in future studies. Additionally, the sub-group analysis of first-line chemotherapy across all Charlson-Deyo score groups showed that, despite an increased percentage of patients with higher Charlson-Deyo scores who did not receive chemotherapy, those who received chemotherapy had better OS in all score groups. This suggests that a higher comorbidity score itself should not automatically deter chemotherapy administration unless there are contra-indications or concerns about serious toxicity. 

Initiation of chemotherapy more than 4 weeks after diagnosis showed better OS in the univariate analysis, indicating that a delay in chemotherapy initiation by a few weeks may not harm OS in metastatic TNBC. This contrasts with non-metastatic TNBC, where a delay in adjuvant chemotherapy initiation ≥30 days after surgery was associated with worse OS and recurrence-free survival rates [36,37]. The better OS associated with surgery or radiation to the distant metastatic site in the univariate analysis may have been due to selection bias, as such patients are likely to be younger, healthier, and have less metastatic burden to be considered for locoregional treatments. The role of these variables could not be assessed in the multivariate analysis due to missing information from some study patients regarding whether they received surgery or radiation to the distant site. Currently, there is no compelling prospective evidence supporting surgery or radiation to distant metastatic sites in TNBC management. The SABR COMET study showed an OS advantage with stereotactic radiation in oligometastatic cancer (only 18 out of the 99 patients had breast cancer), but the NRG BR-00213 study carried out exclusively in breast cancer patients showed no OS benefit [38]. Similarly, randomized controlled data for surgery of the primary tumor have not demonstrated an OS benefit, and there are no randomized trials examining surgery of the distant metastatic sites [39]. 

Previous studies on racial disparities in TNBC OS outcomes have yielded inconsistent results [11,12,40,41,42]. Disparities in comorbidities and care may have contributed to worse OS outcomes for African–Americans in some studies, as no racial differences in TNBC survival were observed when they received similar treatment under comparable conditions [20,40,41]. In our study, OS did not significantly differ among races, indicating that metastatic TNBC behaves biologically similarly across all racial groups and that equitable access to health care is likely to result in similar OS. HER-2 expression level in TNBC does not influence biology or gene expression [43]; hence, HER-2 expression level itself did not influence OS in our study, aligning with findings in other studies [44]. However, with the newer treatment option trastuzumab deruxtecan approved for metastatic HER-2-low (IHC 1+ or IHC 2+ with negative FISH) TNBC in 2022 based on the DESTINY BREAST-04 trial, patients in the future with HER-2-low TNBC are likely to have better OS than those with HER-2-zero TNBC [45]. 

One limitation of our study is that the newer FDA-approved treatments like olaparib/talazoparib (PARP inhibitors), atezolizumab (PD-L1 inhibitor, later withdrawn from the market in 2021), pembrolizumab (PD-1 inhibitor), sacituzumab govitecan (Trop-2 directed antibody–drug conjugate), and trastuzumab deruxtecan (HER-2 directed antibody–drug conjugate), introduced mainly after 2018, have resulted in slightly improved OS for metastatic TNBC, which is not completely reflected in the 2010–2020 data [26,45,46,47,48,49]. While these new treatments demonstrated improved OS in clinical trials, it is important to note that the absolute difference in OS ranged only from 0 to 6 months compared with control arms. In the real world, this OS difference may be even smaller, as patients tend to experience better outcomes in clinical trials than in real-world settings. Our study results will serve as a benchmark for assessing progress in the treatment of metastatic TNBC in future decades. 

Our study’s limitations stem from the NCDB, a hospital-based data collection system that pools data from multiple centers, which may introduce variations in data collection due to possibly distinct data collection standards at each center, alongside potential errors during data abstraction and entry from the medical records. The NCDB also lacks information about relapsed metastatic TNBC, response to first-line therapy, longitudinal treatment data (providing only first-line treatment data without subsequent treatment information), patient-reported outcomes like quality of life, and cause of death. 

## 5. Conclusions

In conclusion, our study on OS in metastatic TNBC is the largest of its kind to comprehensively explore the impact of various factors on OS to better understand survival heterogeneity. These findings empower clinicians to offer personalized prognostic information at diagnosis and tailor treatment strategies. This may involve considering aggressive systemic therapy options versus focusing on comfort/quality of life based on expected prognosis, and considering more frequent scans to assess response to treatment given the short window for therapeutic interventions, especially in those with poor prognostic factors. Our study highlights the clear unmet need for newer therapeutic options to improve outcomes, particularly for those with poor prognostic factors, and can aid in the development of prognostic groups for use in future clinical trials. 

## Figures and Tables

**Table 1:** Univariate analysis of demographic, disease, and treatment characteristics and OS.

	No. of Patients (% of Total Patients)	Median OS in Months (95% CI)	Log-Rank *p*-Value	Hazard Ratio (95% CI)	12-Month Survival Estimates
**Demographic Characteristics**					
**Age**			<0.0001		
19–40	179 (7.9)	15.5 (13.2–18.2)		Reference	0.64 (0.57–0.72)
41–70	1435 (63.1)	15.6 (14.2–16.7)		1.05 (0.87–1.27)	0.58 (0.55–0.60)
71–90	659 (29)	9.6 (8.0–11.4)		1.52 (1.24–1.85)	0.44 (0.41–0.48)
**Race**			0.2033		
Caucasian	1532 (67.4)	13.6 (12.7–14.8)		Reference	0.54 (0.52–0.57)
African–American	638 (28.1)	13.4 (11.9–15.2)		1.02 (0.91–1.14)	0.54 (0.5–0.58)
Other	103 (4.5)	16.9 (12.3–28.3)		0.8 (0.62–1.03)	0.6 (0.51–0.71)
**Charlson-Deyo score**			<0.0001		
0	1732 (76.2)	15.3 (14.3–16.3)		Reference	0.59 (0.56–0.61)
1	339 (14.9)	10.4 (9.1–13.1)		1.22 (1.06–1.41)	0.46 (0.41–0.52)
2	118 (5.2)	6.9 (5.2–9.6)		1.8 (1.47–2.22)	0.35 (0.27–0.45)
3 or more	84 (3.7)	4.6 (2.9–6.9)		2.46 (1.94–3.12)	0.25 (0.17–0.36)
**Clinicopathologic/Cancer Characteristics**					
**Histology**			<0.0001		
Invasive ductal carcinoma	1840 (81)	15.2 (14.1–16.3)		Reference	0.58 (0.56–0.60)
Invasive lobular carcinoma	79 (3.5)	12 (8.5–16.0)		1.23 (0.95–1.60)	0.49 (0.39–0.61)
Adenocarcinoma with metaplasia	64 (2.8)	14.0 (8.9–20.3)		1.0 (0.73–1.4)	0.54 (0.43–0.68)
Adenocarcinoma NOS	105 (4.6)	4.0 (2.9–8.1)		1.89 (1.51–2.37)	0.33 (0.25–0.44)
Other carcinoma	185 (8.1)	6.7 (5.2–9.3)		1.83 (1.55–2.17)	0.35 (0.29–0.43)
**HER-2 IHC expression**					
0	1217 (53.5)	13.1 (12.2–14.5)	0.3099	Reference	0.53 (0.50–0.56)
1+	634 (27.9)	14.2 (12.5–16.1)		0.95 (0.84–1.07)	0.55 (0.51–0.59)
2+	422 (18.6)	14.7 (12.8–17.2)		0.91 (0.79–1.04)	0.57 (0.53–0.62)
**No. of metastatic sites**			<0.0001		
1	1129 (49.7)	19.8 (18.1–22.2)		Reference	0.67 (0.65–0.70)
2	603 (26.5)	11 (9.8–12.3)		1.72 (1.53–1.94)	0.47 (0.43–0.51)
3	328 (14.4)	9.1 (6.9–11.2)		1.99 (1.72–2.30)	0.43 (0.38–0.49)
4	157 (6.9)	4.6 (3.5–5.6)		3.16 (2.62–3.80)	0.24 (0.18–0.32)
5 or 6	56 (2.4)	2.6 (2.2– 5)		4.61 (3.47–6.13)	0.17 (0.10–0.31)
**Site of metastatic involvement (subgroup analysis in those with single metastatic site only after excluding brain metastases)**			0.0001		
LN only	233	24.4 (21.8–33.5)		Reference	0.8 (0.75–0.85)
Bone only	317	18.9 (16.6–24.7)		1.36 (1.08–1.72)	0.67 (0.61–0.72)
Liver only	165	15.6 (13.3–19.4)		1.67 (1.29–2.17)	0.61 (0.54–0.69)
Lung only	286	22.8 (17.6–26.5)		1.23 (0.97–1.56)	0.7 (0.65–0.76)
Other only	83	12.3 (10.2–23.0)		1.85 (1.34–2.55)	0.5 (0.41–0.63)
**Location of metastases**			<0.0001		
Cranial only	45 (2)	11.8 (7.2–19.8)		Reference	0.47 (0.34–0.64)
Cranial + extra-cranial	204 (9)	5.3 (4.5–7.2)		1.7 (1.16–2.49)	0.32 (0.26–0.4)
Extra-cranial only	2024 (89)	14.6 (13.6–15.8)		0.86 (0.60–1.22)	0.57 (0.55–0.59)
**Treatment characteristics**					
**First-line chemotherapy**			<0.0001		
None	613 (27)	2.9 (2.6–3.3)		Reference	0.18 (0.15–0.22)
Single-agent	714 (31.4)	16.4 (14.6–18.4)		0.29 (0.25–0.33)	0.61 (0.58–0.65)
Multi-agent	946 (41.6)	19.9 (18.1–22)		0.23 (0.21–0.26)	0.71 (0.68–0.74)
**Immunotherapy**			<0.0001		
No	1827 (80.4)	11.5 (10.9–12.6)		Reference	0.49 (0.47–0.51)
Yes	446 (19.6)	25.6 (22.3–29.1)		0.49 (0.42–0.56)	0.76 (0.72–0.80)
**Time from Dx to systemic therapy**			<0.0001		
None	579 (25.5)	2.7 (2.4–3.1)		Reference	0.47 (0.39–0.56)
<2 weeks	138 (6.1)	10.8 (9.2–15)		0.34 (0.28–0.43)	0.61 (0.57–0.66)
2–4 weeks	425 (18.7)	15.6 (14.2–16.8)		0.26 (0.23–0.31)	0.71 (0.68–0.73)
>4 weeks	1131 (49.8)	20.6 (19.3–22.6)		0.21 (0.18–0.23)	0.17 (0.14–0.2)
**Surgery of distant site (LN or other site)**			<0.0001		
No	2134 (93.9)	13.1 (12.5–14.1)		Reference	0.53 (0.51–0.55)
Yes	106 (4.7)	29.2 (23.0–NE)		0.50 (0.38–0.66)	0.76 (0.69–0.85)
**Radiation to the distant site**			0.0204		
No	1603 (70.5)	12.6 (11.5–13.6)		Reference	0.51 (0.49–0.54)
Yes	482 (21.2)	14.7 (12.7–17.5)		0.86 (0.76–0.98)	0.57 (0.53–0.61)
**Palliative treatment (to alleviate symptoms)**					
No	1721 (75.7)	14.5 (13.5–15.6)	0.0037	Reference	0.56 (0.54–0.59)
Yes	552 (24.3)	11.3 (10.0–12.9)		1.18 (1.06–1.32)	0.48 (0.44–0.53)

**Table 2:** Sub-group analysis of first-line chemotherapy receipt in each Charlson-Deyo comorbidity score group.

	No. of Patients	Median OS in Months (95% CI)	Log-Rank *p*-Value	Hazard Ratio (95% CI)	12-Month Survival Estimates
**Charlson-Deyo comorbidity score 0;** no. of patients = 1732 (76.2%)					
**First-line chemotherapy**			<0.0001		
None	401	3.3 (2.9–4.0)		Reference	0.22 (0.18–0.27)
Single-agent	550	17.3 (15.5–19.7)		0.31 (0.27–0.36)	0.65 (0.61–0.69)
Multi-agent	781	20.1 (18.4–22.4)		0.26 (0.23–0.3)	0.72 (0.69–0.75)
**Charlson-Deyo comorbidity score 1;** no. of patients = 339 (14.9%)					
**First-line chemotherapy**			<0.0001		
None	111	2.4 (1.7–3.3)		Reference	0.15 (0.09–0.24)
Single-agent	112	13.1 (9.6–17.7)		0.28 (0.21–0.38)	0.51 (0.43–0.62)
Multi-agent	116	19.9 (16.4–29.1)		0.19 (0.14–0.26)	0.7 (0.62–0.79)
**Charlson-Deyo comorbidity score 2;** no. of patients = 118 (5.2%)					
**First-line chemotherapy**			<0.0001		
None	52	2.2 (1.4–3.4)		Reference	0.08 (0.03–0.21)
Single-agent	31	13.2 (10.9–NE)		0.19 (0.11–0.33)	0.57 (0.41–0.77)
Multi-agent	35	16.8 (8.6–25.2)		0.21 (0.13–0.35)	0.54 (0.4–0.74)
**Charlson-Deyo comorbidity score = 3 or more;** no. of patients = 84 (3.7%)					
**First-line chemotherapy**			<0.0001		
None	49	2.2 (1.5–3.7)		Reference	0.1 (0.04–0.24)
Single-agent	21	10.9 (5.3–21.2)		0.3 (0.16–0.54)	0.41 (0.24–0.7)
Multi-agent	14	12.1 (7.5–NE)		0.28 (0.14–0.56)	0.5 (0.3–0.84)

**Table 3:** Multi-variate Cox proportional hazard analysis.

	Hazard Ratio (95% CI)	Overall *p*-Value
**Charlson-Deyo score**		<0.0001
0	Reference	
1	1.23 (1.07–1.42)	
2	1.57 (1.27–1.93)	
3	1.97 (1.55- 2.51)	
**Histology**		<0.0001
Invasive ductal carcinoma	Reference	
Invasive lobular carcinoma	1.04 (0.80–1.35)	
Adenocarcinoma with metaplasia	0.83 (0.60–1.14)	
Adenocarcinoma NOS	1.29 (1.03–1.62)	
Other carcinoma	1.54 (1.29–1.82)	
**No. of metastatic sites**		<0.0001
1	Reference	
2	1.90 (1.69–2.15)	
3	2.16 (1.86–2.5)	
4	3.58 (2.96–4.33)	
5 or 6	4.81 (3.61–6.42)	
**First-line chemotherapy**		<0.0001
None	Reference	
Single-agent or multi-agent	0.28 (0.25–0.32)	
**Immunotherapy**		<0.0001
No	Reference	
Yes	0.56 (0.49–0.65)	

## Data Availability

Data cannot be provided by the authors of this study as the data are from the National Cancer Database. Data can only be requested directly from the National Cancer Database (NCDB).
